# Novel Silsesquioxane-Derived Boronate Esters—Synthesis and Thermal Properties

**DOI:** 10.3390/molecules26144107

**Published:** 2021-07-06

**Authors:** Miłosz Frydrych, Daria Pakuła, Bogna Sztorch, Dariusz Brząkalski, Robert E. Przekop, Bogdan Marciniec

**Affiliations:** 1 Faculty of Chemistry, Adam Mickiewicz University in Poznań, 8 Uniwersytetu Poznańskiego, 61-614 Poznań, Poland; frydrych@amu.edu.pl (M.F.); darpak@amu.edu.pl (D.P.); dariusz.brzakalski@amu.edu.pl (D.B.); 2 Centre for Advanced Technologies, Adam Mickiewicz University in Poznań, 10 Uniwersytetu Poznańskiego, 61-614 Poznań, Poland; rprzekop@amu.edu.pl

**Keywords:** POSS, borasilsesquioxanes, borane, organoboron, heterosilsesquioxanes, hydrosilylation, vinylboranes, thermal decomposition

## Abstract

The functionalization of mono- and octahydrospherosilicate with vinylboranes and allylboranes via hydrosilylation reaction in the presence of a Karstedt’s platinum (0) catalyst is presented. This is the catalytic route to obtain a new class of silsesquioxanes containing boron atoms in their structure in high yields (>90%) and with satisfactory selectivity. The obtained compounds were fully characterized by spectroscopic (^1^H, ^13^C, ^29^Si NMR) and spectrometric methods (MALDI-TOF-MS), as well as thermal analysis (TGA). The obtained compounds were subjected to thermal tests, characterizing the processes of melting, thermal evaporation, sublimation and thermal decomposition.

## 1. Introduction

Silsesquioxanes of the general formula (RSiO_3/2_)_n_ (where n = 6, 8, 10, 12; R = H, alkyl, aryl, halogen, etc.) belong to a wide class of hybrid organosilicon compounds that have both organic and inorganic properties. The well-defined, three-dimensional structure of silsesquioxanes consists of an inorganic, rigid silicon–oxygen core and organic substituents, which can be divided into reactive and inert [1]. Due to their relatively easy synthesis, numerous possibilities of modification, as well as unique physicochemical properties (high thermal stability, low dielectricity, high chemical resistance, biocompatibility, etc.), they are key materials for application in such fields as medicine, biochemistry, catalysis or coordination chemistry [2,3,4,5,6,7]. 

The best known class of these compounds with the greatest applicability potential are fully condensed octasubstituted silsesquioxanes (n = 8), which may contain reactive groups or reactive and inert groups in various molar ratios. They can be easily functionalized by catalytic reactions, e.g., the Heck coupling reaction [8,9,10], silylative coupling [11,12], metathesis [13,14] and others [15,16,17]. 

Hydrosilylation reactions are the most popular methods for obtaining new silsesquioxane derivatives. The reaction mechanism assumes addition of an Si-H bond to unsaturated -C=C- bonds in the presence of transition metal complexes, e.g., platinum, rhodium, cobalt, iridium, ruthenium and others [18,19,20,21,22,23]. Karstedt’s catalyst is one of the most frequently used both on a laboratory and industrial scale due to the possibility of obtaining high yields and high selectivity of the products. Organofunctional silsesquioxanes have been used in many fields, such as electronics, hybrid materials, nanocomposites and space application [24,25].

According to literature reports, there are known examples of functionalization of silsesquioxanes with compounds containing a boron atom in the structure. An example is the work by Kaźmierczak et al., which shows the direct attachment of the boron atom to the cage as a result of dehydrogenative *O*-bororylation of completely and incompletely condensed silsesquioxanes with hydroborates (including pinacoloborate or cathechoborate) [26]. Q. Wang et al. synthesized a silsesquioxane with a phosphorus DOPO group, and then modified it with boric acid. The conducted research proved that modification improved the thermal stability of the filler–polymer system compared to the polymer matrix itself [27]. Another example of the use of borasilsesquioxanes is the work of K. Suenag et al., in which the authors modified silsesquioxane (OctaPhenyl-POSS^®^) with luminescent boro-organic complexes. The obtained product—mechanoluminescent chromium dye—was characterized by improved optical and thermal properties. This was due to the radial arrangement of the substituents in the organosilicon skeleton [28]. Numerous applications of this group of compounds result from the easy functionalization of silsesquioxanes, which enables the control and direction of the properties of the final product. Such compounds have also been used, e.g., as insulating layers [29] or as supports for olefin polymerization catalysts [30,31].

Functionalization of octahydrosilisesquioxane (SS-8H) and monohydrosilsesquioxane (iBu_7_SS-H) by hydrosilylation reactions has been reported in many publications and patents [32,33,34,35,36]. To the best of our knowledge, so far no one has undertaken functionalizing this type of compounds with vinylboranes by catalytic hydrosilylation reaction.

The aim of the work was to synthesize new iBu_7_SS-H and SS-8H spherosilicate derivatives containing functional groups with a boron atom in the structure. The obtained compounds were fully characterized in terms of spectroscopy and thermal stability, which will allow us to determine the potential application.

## 2. Results and Discussion

**Figure 1:**
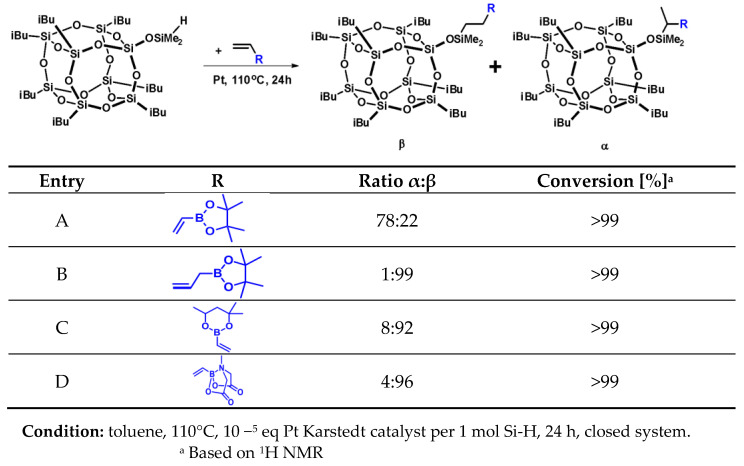
Selectivity and conversion of vinyl boronate hydrosilylation reactions with iBu_7_SS-H.

The choice of the vinyl- and allylboronates applied for this study was made on the basis of the products thereof being potential reagents for further organic group transformations, e.g., Suzuki–Miyaura or Liebeskind–Srogl coupling. Due to their satisfactory stability and commercial availability, boronic esters such as pinacol or MIDA boronates are considered versatile organoboron reagents [37,38,39]. Therefore, we decided to choose unsaturated boronates, being easily commercially available and stable under standard conditions. Examination of ^1^H, ^13^C NMR and ^29^Si NMR spectra, as well as the MALDI-TOF-MS showed full agreement of the products’ structure with the expectancy, as shown in Figure 1. All the isolated products were observed on MALDI-TOF-MS as molecular adducts with a sodium atom.

The results collected in Figure 1 show full conversion of Si-H moiety, confirmed by ^1^H NMR analyses. On the basis of NMR spectroscopy, we determined the ratio of α and *β* isomers in the obtained post-reaction mixtures. The presence of boron atom has a strong electron-withdrawing effect for the double bond of vinylboronic acid pinacol ester, which resulted in the formation of significant amounts of *α*-isomer products for both mono- and octaspherosilicate. At the same time, the products of vinylboronic acid MIDA ester hydrosilylation were formed in an almost *β*-specific manner, due to two reasons, one being the high steric hindrance of MIDA bicyclic moiety, and the second being the electron density transfer from nitrogen to boron atom, which suppresses its electropositive character. For the allyl derivative, an additional carbon atom separating the boron atom from the vinyl moiety prevents this electron-withdrawing effect. The sterical effect of a substituted six-membered ring affected the high *β*-regioselectivity of the obtained product **C** when compared to the five-membered pinacol ring of the product mixture **A**.

**Figure 2:**
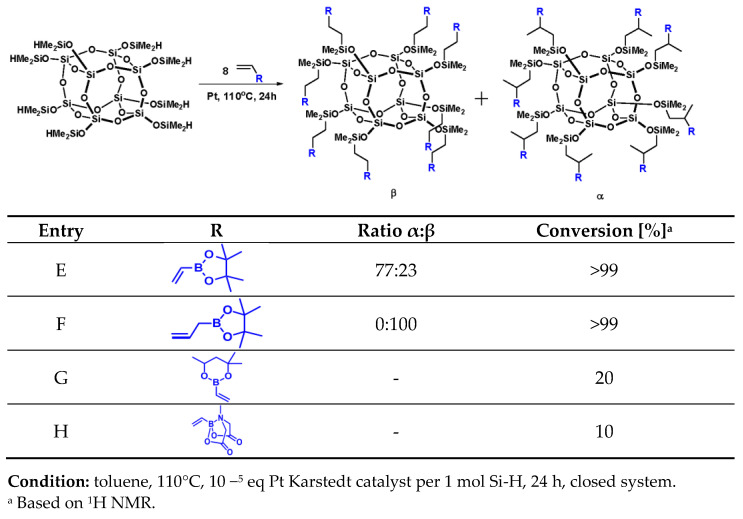
Selectivity and conversion of hydrosilylation reactions with SS-8H.

Positive results of the preliminary tests with iBu_7_SS-H encouraged us to use an octasubstituted derivative, SS-8H, with the same type of boron-containing olefins. General scheme of reaction and the results are collected in Figure 2.

On the basis of the results presented in Figure 2, it can be observed that the reaction leading to the formation of products **E** and **F** proceeded with almost complete conversion of the Si-H moiety (>99%). We observed similar results to the tests with the monosubstituted spherosilicate derivative, such as the electron-withdrawing effect for product **E**, or high *β* product isomer formation for product **F**. For octasubstituted spherosilicates, it was suspected that the steric hindrance of the cyclic MIDA moiety may be highlighted to an even greater degree due to its substitution in as many as eight corners of the cage. However, this explanation is not sufficient to cover the very low conversion observed for products **G** and **H** (10–20%). The NMR spectra of post-reaction mixtures suggest that the boronate reagents were undergoing polymerization instead of hydrosilylation due to high concentration of the vinyl reagent for octasubstituted product. Unfortunately, the experiments on system dilution or temperature lowering did not allow for obtaining the desired products, due to low kinetics of the reaction under such conditions. It could be the explanation for products **G** and **H** being obtained with much lower conversion rates. Both 4,4,6-trimethyl-2-vinyl-1,3,2-dioxaborinane and vinylboronic acid pinacol ester were described in detail in a work concerning Heck coupling [40], where it was proven that under the tested reaction conditions, both reagents are similar in reactivity with a slight advantage in favor of vinylboronic acid pinacol ester, which was explained on the basis of the steric hindrance of both the substrates. Additionally, due to this steric hindrance, these two vinylboronates are characterized by satisfactory stability upon storage. However, as the reactions with monohydrospherosilicase iBu_7_SS-H proceeded quantitatively under the assumed reaction conditions, the steric hindrance of the vinylboronates could not serve as an explanation for the conversion rates of Si-H observed for octahydrospherosilicate SS-H in this study, as the observed rates were as poor as 5–10%.

As the reagents were thoroughly purified before use and the catalytic tests repeated numerous times in closed Schlenk reactors, the chances of reaction issues related to accidental contamination of the reaction mixture were ruled out. Possible vinylboronate polymerization was suspected due to much higher boronate reagent concentration when compared to monospherosilicate analogues (see Section 3.3 and Section 3.4). Therefore, additional experiments were run for octaspherosilicate systems, where both reaction dilution and temperature reduction were tested to suppress the polymerization effect. An additional possibility was platinum-catalyzed, temperature-mediated transesterification between boronate groups, resulting in the curing of reaction components, as high yields of insoluble, glassy matter were produced during reaction.

**Figure 3:**
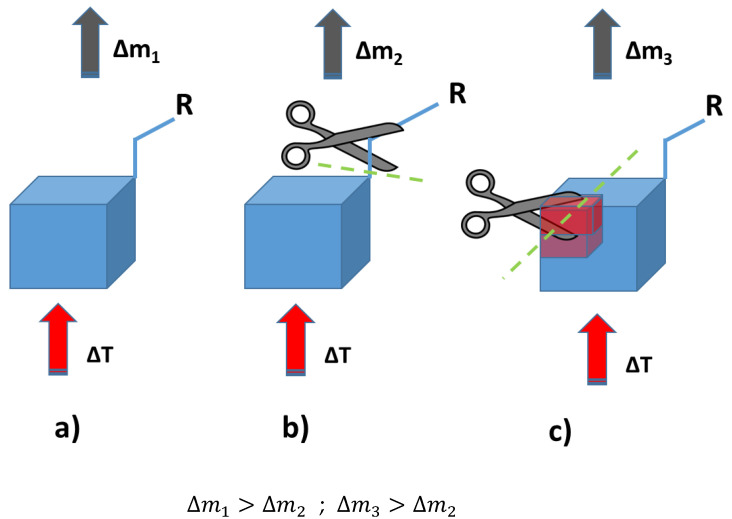
Suggested mechanisms of thermal changes taking place in the synthesized compounds. Route (**a**)—sublimation of compounds; route (**b**)—fragmentation of attached functional groups; route (**c**)—fragmentation of the cube core.

**Figure 4:**
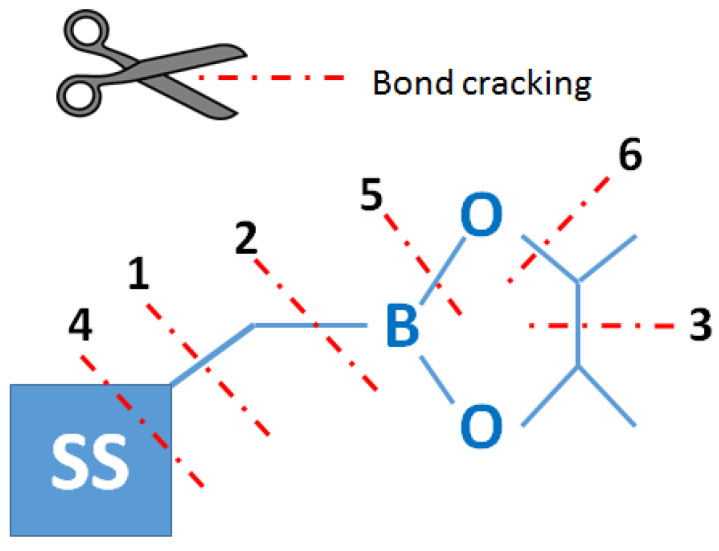
Probability of bonds’ scission based on their energy (based on the energy of a single bond —stabilization effects were not taken into account).

**Figure 5:**
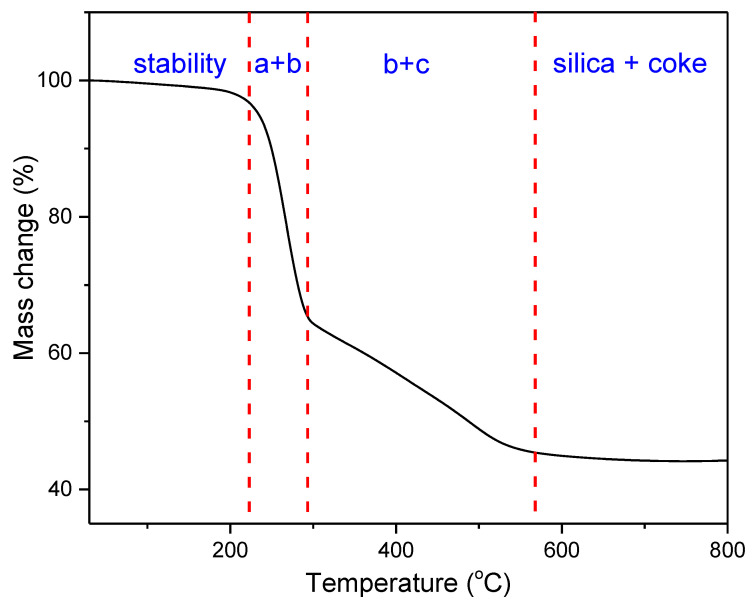
Overview of the complex process of thermal changes of POSS (on the example of iBu7SS-H).

**Figure 6:**
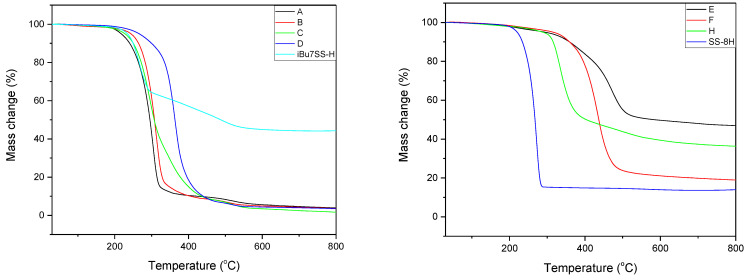
TGA curves of iBu_7_SS-H and SS-8H and its derivatives.

**Figure 7:**
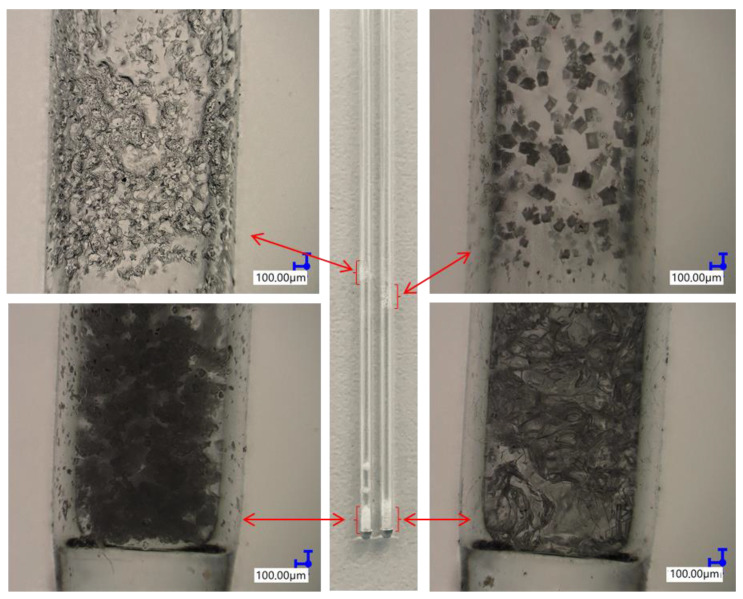
Pictures taken with an optical microscope of capillaries for which both the sublimation process and the thermal decomposition of iBu_7_SS-H and SS-8H derivatives were observed.

Thermogravimetric analysis was performed for all obtained compounds under nitrogen atmosphere. Suggested mechanisms of thermal changes taking place in the synthesized compounds are presented in Figure 3 and Figure 4. The results of the thermogravimetric analysis are presented in Figure 5, Figure 6 and Figure 7. The determination of the silsesquioxanes decomposition mechanism is difficult due to the structural complexity of the discussed compounds [41]. Thermogravimetric measurements allow us to estimate the influence of the modifier (vinylborates) on the overall thermal stability of the compounds obtained. During the process of supplying heat to the sample to POSS-type compounds in an inert gas atmosphere, there may be three major transformations, as shown in Figure 3. The transformation by which the sample passes from the solid phase directly to the gas phase or through phase transformation—melting (not observed on the gravimetric curve) followed by evaporation (a). The course of the gravimetric curve for this process is usually very steep (Figure 5 and Figure 6 SS-8H). Samples whose volatility does not allow for sublimation or evaporation undergo thermal decomposition at temperatures higher than 250–300 °C (Figure 6). The thermal decomposition is related to bond cracking, as can be seen from Table 1, and the Si-C bond occurring near the corner of the diatomaceous spheres (Figure 4) is the most susceptible to breaking, illustrated in Figure 3b as thermal decomposition outside the cage. This mechanism leads to the formation of volatile products which are observed in the gravimetric curve as weight loss in regions above 250 °C. At the same time, at temperatures above 300 °C, the silicon cage bonds may break (Si-O bonds) and form volatile products containing Si atoms (mechanism Figure 3c). It should be noted that for most complex POSS derivatives, the mechanism of thermal gravimetric changes will be a superposition of several effects (Figure 5). For lower temperatures, we will observe a relatively fast sublimation, confirmed by microscopic photos (Figure 7), and at higher temperatures, pyrolysis and release of volatile products or formation of a solid, ceramic residue consisting mainly of SiO_2_ will occur.

In the case of the iBu_7_SS-H derivative (Figure 5), we are dealing with both the sublimation process (area a) and bond-cracking processes (b, c), leading to both the release of volatile products and the formation of SiO_2_-containing residues [43].

Figure 6 shows the thermograms of the obtained substrates and products. Thermal analysis of **A**–**D** products shows the decomposition described by the mechanism of Figure 3c, which consists of the fragmentation of the cage bonds and the formation of silicon-containing volatile products. A detailed analysis of the thermal decomposition of the iBu_7_SS-H substrate is shown in Figure 5. The thermogravimetric curve of the SS-8H substrate is characteristic of the sublimation process. Modification of the compound with vinylborates completely changes the shape of the thermogram, which proves other phase transformations of functionalized products. The highest value of residual masses of the synthesized compounds is present for samples **E** and **H** (47.03 and 36.34% (Table 2)), which indicates the decomposition mechanism presented in Figure 3b, characterized by the breakage of Si-C bonds, which leads to remnants of the cage structure. The thermogravimetric curve shows that sample **F** is transient between the substrate and sample **E**. The residual mass value of 19.02% allows for the conclusion that the sample distribution is complex and indicates loss of functional groups connected with fragmentation of the cage structure.

iBu_7_SS-H and SS-8H were both proven to sublime during heating, as during the measurements of melting point, no melting was observed, but resublimed material was observed on the capillary walls (Figure 7). For compounds **A**, **B**, **C** and **D**, the melting points determined were 114, 96, 72 and 207 °C, respectively. Compounds **E** and **F** were viscous oils at room temperature. Reaction product **H** decomposed at temperatures above 300 °C. The decomposition of reaction product **H** was associated with the color change of the beige compound to red.

## 3. Materials and Methods

### 3.1. Materials

The chemicals were purchased from the following sources:, allylboronic acid pinacol ester, 4,4,6-trimethyl-2-vinyl-1,3,2-dioxaborinane and vinylboronic acid pinacol ester from Tokyo Chemical Industry Co.; vinylboronic acid MIDA ester and Karstedt’s catalyst from Sigma-Aldrich; toluene and acetonitrile from Chempur; and benzene-d_6_ and toluene-d_8_ from Deutero. Vinylboronic acid MIDA ester was dissolved in acetone and then purified by standard chromatography method with a short column filled with silica gel. The eluate was collected and then evaporated under reduced pressure. Toluene was dried over P_2_O_5_, distilled under argon and stored under argon atmosphere in Rotaflo Schlenk flasks over Na/K alloy.

### 3.2. Synthesis of Organosilicon Precursors

Heptaisobutylomonohydrospherosilicate iBu_7_SS-H (1) was synthesized according to the procedure given in the literature [44] with an isolated yield of 91% based on heptaisobutyltrisilanol. Octakishydridooctaspherosilicate SS-8H (2) was synthesized according to the procedure given in the literature [45]. Product was obtained with 95% yield.

### 3.3. General Procedure for Hydrosilylation Tests with Heptaisobutylmonohydrospherosilicate

All hydrosilylation reactions were conducted under argon atmosphere in 25 mL high-pressure Schlenk reactors equipped with a Rotaflo stopcock and magnetic stirring bars. In a typical procedure, a Schlenk’s reactor was charged with 0.056 mmol (50 mg) of iBu_7_SS-H, 3 mL of toluene and 0.056 mmol of olefin. Karstedt’s catalyst solution (10−5 eq Pt/mol Si-H) was added. The reaction mixture was set at 110°C for 24 h. After removal of the solvent under reduced pressure, ^1^H NMR analysis was run to measure conversion rate and product selectivity.

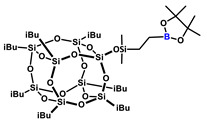



**^1^H NMR** (400 MHz, CDCl_3_): δ (ppm) = 1.93–1.78 (m, 63H, iBu), 1.24 (s, 12H, β isomer pinacol Me), 1.22 (s, 12H, α isomer pinacol Me), 1.06 (d, J = 7.3 Hz, 3H, α isomer BCH(**CH_3_**)Si), 0.95 (d, J = 6.6Hz, 42H, iBu), 0.62–0.58 (m, 14H, iBu), 0.47 (q, 1H, α isomer B**CH**(CH_3_)Si), 0.15 (d, J = 8.8Hz, α isomer SiMe_2_), 0.08 (s, β isomer SiMe_2_);

**^1^H NMR** (400 MHz, C_6_D_6_): δ (ppm) = 2.16–2.01 (m, iBu), 1.40 (d, J = 7.3 Hz, α isomer SiCH(**CH_3_**)Si), 1.23 (t, J = 6.2 Hz, α isomer B**CH_2_**CH_2_Si), 1.10–1.05 (m, pinacol Me; iBu), 0.98–0.90 (m, β isomer BCH_2_**CH_2_**Si), 0.85–0.81 (m, iBu), 0.80–0.74 (q, J = 7.3, α isomer Si**CH**(CH_3_)Si), 0.44 (d, J = 10.4 Hz, α isomer SiMe_2_), 0.26 (s, β isomer SiMe_2_);

**^13^C NMR** (101 MHz, CDCl_3_): δ (ppm) = 83.0, 82.0 (pinacol ring), 25.9 (iBu), 25.1, 25,0 24.8 (pinacol Me), 24.0, 23.9, 22.6, 22.5 (iBu), 8.3 (B**CH**(CH_3_)Si), 0.11, −0.9 (SiMe_2_);

**^13^C NMR** (101 MHz, C_6_D_6_): δ (ppm) = 82.8 (pinacol ring), 26.0 (iBu), 25.1 (pinacol Me), 25.0, 24.9, 24.4, 23.1, 23.0, 22.9 (iBu), 11.0 (B**CH_2_**CH_2_Si), 8.7 (B**CH**(CH_3_)Si), 1.4, 0.4, −0.4, ((BCH_2_**CH_2_**Si), SiMe_2_ α isomer, SiMe_2_ β isomer);

**^29^Si NMR** (79,5 MHz, C_6_D_6_): δ (ppm) = 12.77 (β isomer SiMe_2_), 11.92 (α isomer SiMe_2_), −66.67, −66.71, −67.55 (cage), −109.32 (β isomer SiMe_2_ SiO_4_), −109.66 (α isomer SiO_4_).

**MALDI-TOF-MS: [M]+Na^+^: 1067.3804**

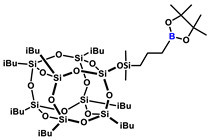



**^1^H NMR** (400 MHz, C_6_D_6_): δ (ppm) = 2.14–2.02 (m, 7H, iBu), 1.80–1.72 (m, 2H, B**CH_2_**CH_2_CH_2_Si), 1.09–1.05 (m, 54H, pinacol Me, iBu), 1.05–1.02 (m, 2H, BCH_2_**CH_2_**CH_2_Si), 0.87–0.81 (m, 16H, BCH_2_CH_2_**CH_2_**Si, iBu), 0.25 (s, 6H, SiMe_2_);

**^13^C NMR** (101 MHz, C_6_D_6_): δ (ppm) = 82.7 (pinacol ring), 26.0 (iBu), 25.1 (pinacol Me), 24.4, 23.1, 23.0, 22.9 (iBu), 21.5 (B**CH_2_**CH_2_CH_2_Si), 18.3 (BCH_2_**CH_2_**CH_2_Si), 1.4 (BCH_2_CH_2_**CH_2_**Si);

**^29^Si NMR** (79,5 MHz, C_6_D_6_): δ (ppm) = 11.32 (SiMe_2_), −66.69, −66.72, −67.56 (cage), −109.41 (SiO_4_).

**MALDI-TOF-MS: [M] + Na^+^: 1081.3910**

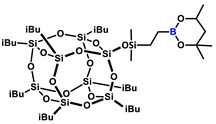



**^1^H NMR** (400 MHz, C_6_D_6_): δ (ppm) = 3.92–3.87 (m, 1H, C(CH_3_)**H**, borane ring), 2.21–2.03 (m, 7H, iBu), 1.41 (d, 1H, C(**CH_3_**)H, borane ring), 1.14–0.95 (m, 50H, C**H_a_H_b_**, C**(CH_3_)_2_**, borane ring, iBu), 0.86–0.81 (m, 14H, iBu), 0.43 (t, 2H, Si**CH_2_**-), 0.24 (s, 6H, SiMe_2_);

**^13^C NMR** (101 MHz, C_6_D_6_): δ (ppm) = 70.3 (**C**(CH_3_)_2_, borane ring), 64.6 (**C**(CH_3_)H, borane ring), 46.1 (**C**H_a_H_b_, borane ring), 31.5, 28.2 (C**(CH_3_)_2_**, borane ring), 26.0, 24.4 (iBu), 23.5 (C(**CH_3_**)H), 23.1, 23.0 (iBu), 11.2 (B**CH_2_**CH_2_Si), 8.8 (BCH_2_**CH_2_**Si), 0.7 (SiMe_2_);

**^29^Si NMR** (79,5 MHz, C_6_D_6_): δ (ppm) = 12.54 (SiMe_2_), −66.47, −66.74, −67.54 (cage), −109.61 (SiO_4_).

**MALDI-TOF-MS: [M] + Na^+^: 1067.3794**

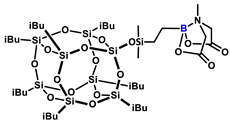



**^1^H NMR** (400 MHz, CDCl_3_): δ (ppm) = 3.86 (d, J = 16.3 Hz, 2H, -C(O)CH_2_), 3.67 (d, J = 16.3 Hz, 2H, -C(O)CH_2_), 2.90 (s, 3H, NMe), 1.93-1.78 (m, 7H, iBu), 0.97-0.94 (m, 42H, iBu), 0.61–0.58 (m, 14H, iBu), 0.54-0.48 (m, 4H, B**CH_2_CH_2_**Si), 0.11 (s, 6H, SiMe_2_);

**^13^C NMR** (101 MHz, CDCl_3_): δ (ppm) = 167.0 (C=O), 62.4 (-C(O)CH_2_-), 45.6 (NMe), 25.8, 24.0, 22.6 (iBu), 10.4 (SiCH_2_-) −0.7 (SiMe_2_);

**^29^Si NMR** (79,5 MHz, Tol-d8): δ (ppm) = 12.80 (SiMe_2_), −66.69, −67.53, −67.56 (cage), −109.04 (SiO_4_).

**MALDI-TOF-MS: [M] + Na^+^: 1097.3337**

### 3.4. General Procedure for Hydrosilylation Tests with Octakishydridooctaspherosilicate

All hydrosilylation reactions were conducted under argon atmosphere in 25 mL high-pressure Schlenk reactors equipped with a Rotaflo stopcock and magnetic stirring bars. In a typical procedure, a Schlenk’s reactor was charged with 0.0246 mmol (25 mg) of octakishydridooctaspherosilicate SS-8H, 3 mL of toluene and 0.197 mmol of olefin. Karstedt’s catalyst solution (10 ^−5^ eq Pt/mol Si-H) was added. The reaction mixture was carried out at 110 °C for 24 h. After removal of the solvent under reduced pressure, ^1^H NMR analysis was run to measure conversion rate and product selectivity.

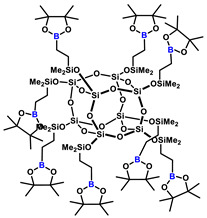



**^1^H NMR** (400 MHz, CDCl_3_): δ (ppm) = 1.23 (s, β isomer pinacol Me), 1.20 (s, α isomer pinacol Me), 1.06 (d, J = 7.2Hz, α isomer SiCH(**CH_3_**)B), 0.90-80 (m, 2H, SiCH_2_**CH_2_**B), 0.77–0.60 (m, 2H, Si**CH_2_**CH_2_B), 0.51 (q, J = 7.2 Hz, α isomer Si**CH**(CH_3_)B), 0.17 (s, β isomer SiMe_2_), 0.11 (d, J = 8.8 Hz, α isomer SiMe_2_);

**^1^H NMR** (400 MHz, Tol-d8): δ (ppm) = 1.41–1.39 (m, α isomer SiCH(**CH_3_**)B), 1.16–1.10 (m, pinacol Me), 1.05–0.91 (m, 2H, Si**CH_2_**CH_2_B), 0.82–0.79 (m, 1H, Si**CH_2_**CH_2_B), 0.45 (d, J = 8.8 Hz, α isomer SiMe_2_), 0.32 (s, β isomer SiMe_2_);

**^13^C NMR** (101 MHz, Tol-d8): δ (ppm) = 82.7, 82.6 (pinacol ring), 25.0 (pinacol Me), 10.8 (B**CH_2_**CH_2_Si), 8.8 (B**CH**(CH_3_)Si), 1.4 (BCH(**CH_3_**)Si, 0.6–(−0.7) ((BCH_2_**CH_2_**Si), SiMe_2_ α isomer, SiMe_2_ β isomer);

**^29^Si NMR** (79,5 MHz, Tol-d8): δ (ppm) = 13.36 (β isomer SiMe_2_), 12.53 (α isomer SiMe_2_), −108.84 (core).

**MALDI-TOF-MS: [M] + Na^+^: 2248.9294**

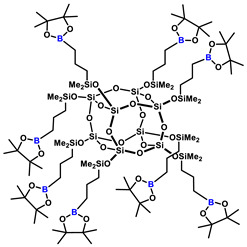



**^1^H NMR** (400 MHz, Tol-d8): δ (ppm) = 1.75–1.67 (m, 16H, B**CH_2_**CH_2_CH_2_Si), 1.11 (s, pinacol Me), 1.09–1.01 (m, 16H, BCH_2_**CH_2_**CH_2_Si), 0.87–0.83 (m, BCH_2_CH_2_**CH_2_**Si), 0.29 (s, 48H, SiMe_2_);

**^13^C NMR** (101 MHz, Tol-d8): δ (ppm) = 82.6 (pinacol ring), 25.0 (pinacol Me), 21.3 (B**CH_2_**CH_2_CH_2_Si), 18.2 (BCH_2_**CH_2_**CH_2_Si), 1.4 (BCH_2_CH_2_**CH_2_**Si), 0.01 (SiMe_2_);

**^29^Si NMR** (79,5 MHz, Tol-d8): δ (ppm) = 12.31 (SiMe_2_), –108.43 (core).

**MALDI-TOF-MS: [M] + Na^+^: 2385.0117**

### 3.5. Analytical Methods

The ^1^H NMR spectra were recorded on a Bruker Ultrashield 300 MHz. The ^13^C and ^29^Si NMR spectra were recorded on a Bruker Ascend 400 MHz operating at 101 and 79 MHz, respectively. Benzene-d_6_ was used as a solvent.

MALDI-TOF mass spectra were recorded on a UltrafleXtreme mass spectrometer (Bruker Daltonics), equipped with a SmartBeam II laser (355 nm) in the 500–4000 m/z range, and 2,5-dihydroxybenzoic acid (DHB, Bruker Daltonics, Bremen, Germany) served as the matrix.

Thermogravimetry (TG) was performed using a NETZSCH 209 F1 Libra gravimetric analyzer (Selb, Germany). Samples of 2 ± 0.2 mg were placed in Al_2_O_3_ crucibles. Measurements were conducted under nitrogen (flow of 20 mL/min) in the range of 50–800 °C and a 20 °C/min heating rate.

## 4. Conclusions

Novel boron-containing silsesquioxane derivatives of mono- and octaspherosilicate were obtained by Karstedt’s complex-catalyzed hydrosilylation of various unsaturated boronates. Products were fully characterized by spectroscopic methods that confirmed the structures and purity of the obtained compounds. The chosen boronates are commercially available and shelf-stable, and the silsesquioxane precursors obtainable via well-described synthetic procedures (alternatively, also commercially available). Presumed mechanisms of thermal decomposition of silsesquioxane compounds were determined and characterized for the obtained derivatives. The obtained compounds may play a role as interesting synthons for the preparation of organosilicon hybrid materials or other silicon-containing precursors. This is the first available literature report which analyzed the thermal decomposition of silsesquioxanes containing a boron atom in the structure.

## Figures and Tables

**Table 1:** Bond energy (according to reference [42]).

	Bond	Bond Entalphy kJ/mol
1	Si-C	435
2	B-C	448
3	C-C	607
4	Si-O	798
5	B-O	806
6	C-O	1076

**Table 2:** Summary of the data from the conducted measurements: the beginning of the decomposition process (onset).

Sample Name	5% Mass Loss (°C)	10% Mass Loss (°C)	Temperature at the Maximum Rate of Mass Loss (°C)	Onset Temperature (°C)	Residual Mass 800C (%)
iBu_7_SS-H	234.3	249.6	268.4	241.3	44.23
A	217.7	240.6	304.2	270.1	3.89
B	244.4	264.6	312.4	284.6	3.62
C	228.0	250.3	296.1	267.0	1.67
D	266.6	299.9	363.9	337.6	3.47
SS-8H	220.4	234.1	270.9	240.4	13.92
E	294.1	359.6	359.1	410.5	47.03
F	315.6	359.1	359.6	390.1	19.02
H	288.1	315.9	315.9	311.1	36.34

## Data Availability

All the data used for the preparation for manuscript has been attached to the supplementary information.
